# Live Vaccines Have Different NK Cells and Neutrophils Requirements for the Development of a Protective Immune Response Against Tuberculosis

**DOI:** 10.3389/fimmu.2020.00741

**Published:** 2020-04-22

**Authors:** Ana Paula Junqueira-Kipnis, Monalisa Martins Trentini, Lázaro Moreira Marques Neto, André Kipnis

**Affiliations:** ^1^Laboratory of Immunopathology of Infectious Disease, Department of Biosciences and Technology, Tropical Institute of Pathology and Public Health, Federal University of Goiás, Goiânia, Brazil; ^2^Laboratory of Molecular Bacteriology, Department of Biosciences and Technology, Institute of Tropical Pathology and Public Health, Federal University of Goiás, Goiânia, Brazil

**Keywords:** protection, TB vaccine, DC, NK depletion, neutrophil depletion

## Abstract

It has been shown that neutrophils drive NK cells to activate DCs while NK cells regulate neutrophils survival. In response to mycobacteria, NK cells proliferate and produces IFN-γ, that appears to regulate the neutrophilic inflammatory responses to both *M. tuberculosis* infection and BCG vaccination. Although the role of neutrophils in the immune response to tuberculosis is a matter of debate, neutrophils were shown to be crucial to induce specific response against mc^2^-CMX vaccine. The objective of this study was to investigate the interplay between NK cells and neutrophils in regard to the development of a protective immune response against *M. tuberculosis*. Depletion of NK cells during vaccination did not alter the total number of neutrophils or DCs, but reduced the number of activated DCs, thus reducing the generation of Th1 specific immune responses and the protection conferred by mc^2^-CMX and BCG vaccines. However, only in mc^2^-CMX vaccination that neutrophil depletion interfered with the NK cell numbers and protection. In conclusion, it was shown that only when both NK and neutrophils were present, specific Th1 response and protection was achieved by mc^2^-CMX vaccine, while neutrophils although activated upon BCG vaccination were not necessary for the induced protection.

## HIGHLIGHTS

- Vaccination with BCG or mc^2^-CMX increased the levels of activated neutrophils and NK cells.
- BCG and mc^2^-CMX immunization generated cytotoxic and pro-inflammatory NK subsets.
- NK depletion altered the activation of DCs, specific Th1 response generation and protection against TB generated by mc^2^-CMX and BCG.
- Neutrophil depletion reduced the NK generated by mc^2^-CMX, and also reduced the Th1 response generation and protection against TB.

## Introduction

BCG is the only approved vaccine against tuberculosis (TB). TB is a global disease affecting more than 10.4 million people every year. Although BCG protects against miliary TB in children, the protection is varied among adults. Therefore, at least 14 new vaccine candidates against TB are being evaluated in clinical trials but so far, to our knowledge, none of them were approved to substitute or boost BCG responses (1, 2). Regarding Mtb vaccine responses, NK cells were recently shown to be important for the induction of the Th1 responses to the BCG vaccination as well as presented memory like responses (3–5). NK cells were also shown to be important for a specific immune response against Mtb subunit vaccine (6).

Natural killer (NK) cells are cytotoxic cells detected rapidly after infection (7, 8). NK cells produce cytokines, mainly IFN-γ, although they may also produce other proinflammatory (TNF-α) and immunosuppressive (IL-10) cytokines and chemokines such as CXCL8, important for neutrophil recruitment (9). The production of IFN-γ by NK cells aids the T cell response, activates dendritic cells (DCs) and stimulates naive T cells differentiation into a Th1 phenotype (10–14). Additionally, NK cells promote the activation/maturation of DCs through the production of cytokines such as TNF-α (which increase the expression of costimulatory molecules) and in synergy with IFN-γ, contributes to the production of IL-12) (12, 15). The observation that cells from RAG (Recombinase-1) knockout mice could transfer protection to naïve mice (16) and that NK cells from primates infected with simian immunodeficiency virus (SIV) killed only DCs that were infected with the related SIV strain (5, 17) suggests that NK cells can also acquire specific memory phenotype in response to hapten-carrier antigen or to infection.

NK cells act against *Mycobacterium tuberculosis* (Mtb) infection by inducing apoptosis of infected cells through the interaction of the tumor necrosis factor receptor (TNFR) family (18). *In vivo* studies showed that, in a murine model of infection, NK cells proliferate and migrate to the lungs and produce IFN-γ among other cytokines, which contributes to the generation of specific immune response to Mtb (7, 19). In addition, FENG et al. have shown that mouse infection with Mtb in the absence of IL-12 or IFN-γ, besides to increased susceptibility, resulted in an exacerbated neutrophilic inflammatory reaction, thus indicating that IFN-γ produced by NK cells regulate the neutrophil response to Mtb infection.

Studies of interactions between neutrophils and NK cells in humans have shown that neutrophils stimulated by the TLR (Toll-Like Receptors) instruct NK cells to activate DCs (20) and inversely NK cells regulate neutrophils survival, driving apoptosis, and preventing tissue damage due to over activation (21–23). Neutrophils have also been associated with NK cells maturation in several organs, moreover, in the absence of neutrophils, NK cells were hyper responsive and inflammatory (14). Thus, it appears that NK and neutrophils might interact with each other favoring a modulated immune response against pathogens (19, 24).

The protection mechanisms of a new vaccine need to be very well-understood in pre-clinical assays before being moved to human trials. We have developed a recombinant live vaccine, mc^2^-CMX, composed of recombinant *Mycobacterium smegmatis* expressing CMX fusion protein (composed of Ag85C; MPT-51 and HspX antigens) (25, 26) that was able to induce high numbers of Th1 (TCD4^+^IFN-γ^+^) and Th17 (TCD4^+^IL-17^+^) cells, which culminated in superior protection than BCG against Mtb. Neutrophils were shown to participate in the induction of these specific immune responses to mc^2^-CMX vaccine, once in the absence of these cells the specific immune response to CMX vaccine was abolished (27). Whereas, the interaction between neutrophils and NK cells may be important mediators of specific immune response, it was hypothesized that NK cells could aid neutrophils function. Therefore, the objective of this work was to evaluate the *in vivo* effect of NK cells and neutrophils in the induction of specific and protective responses to mc^2^-CMX and BCG vaccines against *M. tuberculosis*.

## Methods

### Animals

Six weeks old C57BL/6 female mice, specific-pathogen-free from CEMIB/UNICAMP were housed in ABSL-2 racks fitted with HEPA-filtered air intake and exhaust system. The animals were maintained in controled temperature (20–24°C) and relative humidity (40–70%), and a 12-h light/dark cycle, according to recommendations of the Brazilian National Council for Animal Experimentation (Conselho Nacional de Controle de Experimentação Animal- CONCEA). The protocols used in this study were approved by the Ethics Committee on the Use of Animals of the Federal University of Goiás (Protocol number: 027/14).

### Vaccines

The mc^2^ and mc^2^-CMX vaccines used in this work were produced according to Junqueira-Kipnis et al. (25). The vaccines were diluted in PBS 0.05% Tween 80 (10^8^ CFU/mL), and animals were subcutaneously vaccinated twice with 100 μL/animal in a 15 days interval. BCG Moreau vaccine (10^7^ CFU/mL; 100 μL/animal) was administered once subcutaneously as the control.

### Immunization Design

Mice were vaccinated subcutaneously with mc^2^ (10^7^ CFU/mouse), mc^2^-CMX (10^7^ CFU/mouse), BCG (10^6^ CFU/mouse) or saline. Two, five, and seven days after the last immunization, spleen, axillary lymph nodes and the skin and accessory tissues at the immunization site were collected for histological and cytometry analyses.

### Single Cell Suspension From Spleen, Lymph Nodes and *in-situ* Tissues

Cell preparation and cytometry analyses were done as described by Junqueira-Kipnis et al. (25) and da Costa et al. (28). Briefly, mice were euthanized and the lymph nodes, spleen and tissue at the site of vaccine injection were collected. Spleens and lymph nodes were prepared as single-cell suspensions using 70-μm cell strainers (BD Biosciences), and the cells were resuspended with RPMI medium. Erythrocytes were lysed with lysis solution (0.15 M NH_4_Cl, 10 mM KHCO_3_), and the cells were washed and resuspended with RPMI supplemented with 10% fetal calf serum, 0.15% sodium bicarbonate, 1% L-glutamine (200 mM; Sigma-Aldrich, Brazil), and 1% non-essential amino acids (Sigma-Aldrich). Cells were counted in a Neubauer chamber, and the concentration were adjusted to 1 × 10^6^ cells/mL. The *in-situ* tissue was digested with DNAse IV (30 μg/mL; Sigma-Aldrich) and collagenase III (0.7 mg/mL; Sigma-Aldrich Brazil) for 30 min at 37°C. The digested tissue was prepared as single-cell suspensions using 70-μm cell strainers and subjected to erythrocyte lysis. The cells were washed and resuspended with RPMI, and the concentrations were adjusted to 1 × 10^6^ cells/mL.

### Evaluation of the Number of Neutrophils and NK Cells Induced by the Vaccine

Briefly, 10^6^ cells obtained as described previously, were distributed in a 96-well plate, labeled with the antibodies: FITC-anti-CD3 (clone 145-2C11); PE-anti-CD8 (clone 53-6.7); PE-anti-CD27 (clone LG.7F9); PERCP-anti-CD11b (clone M1 / 70); APC-anti-GR1 (clone RB6-8C5), and incubated for 30 min. Afterward, the cells were washed with PBS containing 0.1% sodium azide and fixed with Perm Fix (BD PharMingen). Subsequently, cell acquisition of 50,000 events per sample was performed in a BD FACSVerse™ flow cytometer (Universidade Federal de Goiás, Faculdade de Veterinária e Zootecnia), and the acquired data were analyzed using FlowJo 9.0 software.

### Quantification of NK-IFN-γ^+^ and Neutrophils TNF-α^+^ Cells

Briefly, the cells were incubated with 3 mM monensin (eBioscience) for 4 h at 37°C in a 5% CO_2_ incubator. Subsequently, the cells were labeled with FITC-anti-CD3 (clone 145-2C11) and APC-anti-NK1.1 (clone PK136), APC-anti-GR1 (clone RB6-8C5) for 30 min. After that, cell suspensions were washed with PBS containing 0.1% sodium azide, and fixed and permeabilized with Perm Fix/Perm wash and further incubated with PE-anti-IFN-γ (clone XMG1.2) and PERCP-anti-TNF-α (clone MP6-XT22) for 30 min. After washing with Perm wash (BD PharMingen), the cell suspensions were fixed with Perm Fix. Cell acquisition of at least 50,000 events per sample with minimum 10,000 events in the NK cell gate was performed in a BD FACSVerse™ flow cytometer (Universidade Federal de Goiás, Faculdade de Veterinária e Zootecnia), and the acquired data were analyzed using FlowJo 9.0 software.

### *In vivo* NK Cells Depletion

C57BL/6 mice were treated every 5 for 30 days by intraperitoneal route with 100 μg of anti-asialo-GM1 (WAKO). One day after the first depletion treatment with anti-asialo-GM1 (WAKO), mice were immunized subcutaneously with mc^2^-CMX (10^7^ CFU/animal), mc^2^ (10^7^ CFU/animal), BCG (10^6^ CFU/animal) or saline. Fifteen days after the first immunization mice which received mc^2^-CMX or mc^2^ were boosted with the respective vaccine. Fifteen days after the last immunization, the spleen, axillary lymph nodes and lungs were collected for evaluation of specific immune response induced by vaccination.

### Evaluation of DCs and Neutrophils

For evaluation of DCs and neutrophils, spleen, axillary lymph node, and lung cells were collected and processed as described above. Cells suspensions were labeled with FITC-anti-CD11b (clone M1/70), PE-anti-CD11c (clone N418), PerCP-anti-MHCII (clone M5/114.15.2), and APC-anti-GR1 (clone RB6-8C5) for 30 min all from Ebioscience™. The cells were then washed with PBS containing 0.1% sodium azide, then fixed with Perm Fix (BD PharMingen). 50,000 events were acquired in the BD FACSVerse™ flow cytometer and the data were analyzed with FlowJo 9.0 software. After excluding doublets, cells were gated using the size and granularity of monocytes (FSC-A vs. SSC-A), then DC cell populations were analyzed for specific markers.

### Evaluation of CMX Specific Immune Responses

10^6^ cells from the spleen, axillary lymph nodes, and lungs were plated in 24 well culture plates, stimulated with ConA (1 μg/mL), CMX (10 μg/mL) or without stimulus (medium) and incubated for 4 h in a 5% CO_2_ chamber at 37°C. Then, the plates were incubated for another 6 h with 3 mM monensin (eBioscience). After that, the cells were labeled with FITC-anti-CD4 (GK1.5 clone) for 30 min. Subsequently, the labeled cells were fixed with Perm Fix (BD Cytofix/Cytoperm Kit) for 20 min, and their membranes were permeabilized with Perm wash (BD Cytofix/Cytoperm Kit) for 20 min. After this step, the cells were labeled with the intracellular antibodies: APC-anti-IFN-γ (clone XMG1.2) incubated for 30 min and fixed with Perm Fix. After labeling the cells, 50,000 events were acquired in the Biosciences BD FACSVerseTM flow cytometer and the data were analyzed with FlowJo 9.0 software.

### Mtb Infection

Frozen Mtb aliquots (H37Rv) were diluted in PBS 0.05% Tween 80 to a final concentration of 10^6^ CFU/mL. Mice were challenged 30 days after the last vaccination with 10^5^ CFU/animal, via the intravenous route (lateral tail vein).

The infecting dose was determined by plating the lung homogenate of one mouse per group, 1 day after infection with Mtb. The homogenate was diluted in PBS 0.05% Tween 80 and overlaid on plates containing 7H11 medium supplemented with OADC.

Thirty days after Mtb challenge, the right and medial right lung lobes were collected aseptically, processed, and distributed on plates containing 7H11 medium supplemented with OADC. After distributing the dilutions, the plates were incubated for 21 days at 37°C, and then CFU quantified.

### Evaluation of Neutrophil and NK Interaction

C57BL/6 mice received 100 μg of anti-asialo-GM1 (WAKO) at day −1, by intraperitoneal route; or 200 μg of anti-mouse Ly6G antibodies (BioXcell; clone 1A8) at day −1 and day 2, by intraperitoneal route. At day 0 mice were immunized subcutaneously with mc^2^-CMX (10^7^ CFU/animal), BCG (10^6^ CFU/animal) or saline. Five days after the immunization the spleen, axillary lymph nodes and lungs were collected for evaluation of neutrophils or NK cells responses induced by vaccination.

### Statistical Analysis

The results were tabulated in Excel (version 14.3.4, 2011) and Prism 4 software (Graphpad Software 4.0). The difference between groups was assessed by ANOVA followed by Mann-Whitney test. Differences with *p* < 0.05 were considered as statistically significant.

## Results

### Immunization With mc^2^-CMX Vaccine Induces the Migration of Neutrophils to the Site of Immunization

**Figure 1:**
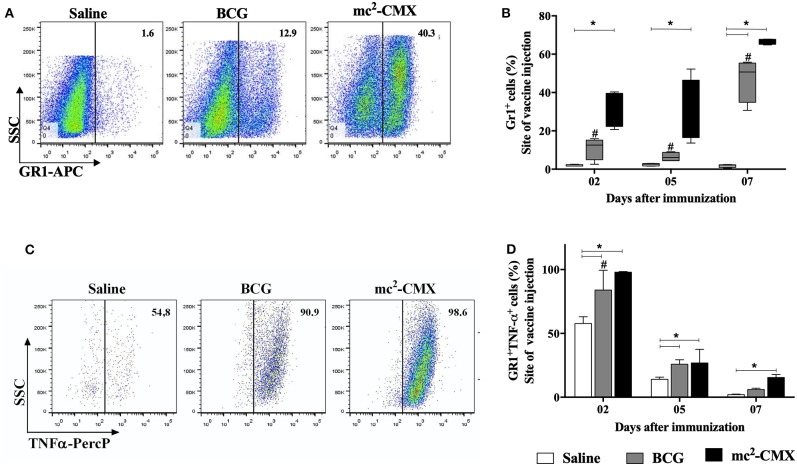
Evaluation of pro-inflammatory neutrophils at site of mc^2^-CMX immunization site. C57BL/6 mice (*N* = 5) were immunized with saline, BCG (10^7^ CFU/mL) or mc^2^-CMX (10^8^ CFU/mL), and after 2, 5, and 7 days, the inflammatory lesions induced by vaccination were collected and processed for neutrophil evaluation by flow cytometry. **(A)** Representative dot plots with neutrophil gates at 2 days post infection. **(B)** Percentage of neutrophils (GR-1^+^). **(C)** Representative dot plots of neutrophil TNF-α^+^ gates (GR-1^+^ TNF-α^+^ found at the site of vaccination at 2 days post infection. **(D)** Percentage of neutrophils TNF-α^+^. *Statistical difference between vaccinated and saline groups (*p* < 0.05). ^#^Statistical difference between BCG and mc^2^-CMX groups (*p* < 0.05).

Although we have previously observed the presence of neutrophils at the site of vaccination with mc^2^-CMX and demonstrated their importance for the induction of specific Th1 and Th17 immune responses (27), the demonstration whether those neutrophils produced any cytokines that could influence the general inflammatory outcome was not investigated. Thus, the presence of TNF-α^+^ neutrophils at the site of vaccination with mc^2^-CMX was evaluated after 2, 5, and 7 days. It can be seen in Figures 1A,B that the percentage of neutrophils, following vaccination with mc^2^-CMX, presented a huge increase starting as soon as 2 days post infection. BCG vaccination presented significant neutrophils migration only at 7 days post infection when compared to the saline injected skin. In addition, the percentage of neutrophils (GR-1) that were TNF-α^+^ peaked 2 days after vaccinations with mc^2^-CMX and BCG while mc^2^-CMX vaccination remained with increased levels at 7 days post infection when compared to saline and BCG (Figures 1C,D). These results indicate that neutrophils migrating to both the mc^2^-CMX or BCG vaccination sites were able to produce TNF-α. However, the mc^2^-CMX vaccine induced significantly higher neutrophil migration and activation at the immunization site.

**Figure 2:**
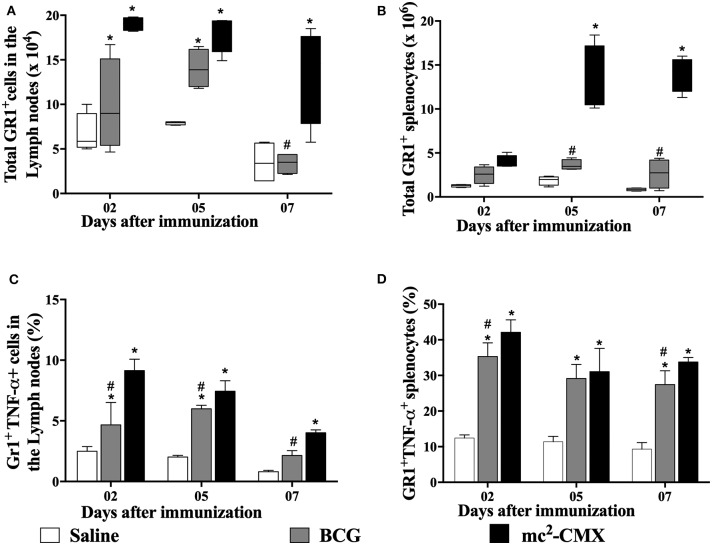
Evaluation of neutrophils in draining secondary lymphoid organs. C57BL/6 mice (*N* = 5) were immunized with saline, BCG (10^7^ CFU/mL) or mc^2^-CMX (10^8^ CFU/mL), and after 2, 5, and 7 days, axillary lymph nodes **(A,C)** and spleen **(B,D)** were collected and processed to evaluate the number of neutrophil **(A,B)** and its activation status **(C,D)**. *Statistical difference between vaccinated and saline groups (*p* < 0.05). ^#^Statistical difference between BCG and mc^2^-CMX groups (*p* < 0.05).

Since mc^2^-CMX vaccine induced an increase in the number neutrophils at the site of vaccination, we questioned whether those neutrophils could be drained to the secondary lymphoid organs (draining lymph nodes and spleen). Although we are not sure that the neutrophils found in those organs were from the mc^2^-CMX or BCG vaccination site, similar increase of neutrophils was observed in both organs (Figures 2A,B). However, when verifying the percentage of TNF-α^+^ neutrophils, only the mc^2^-CMX vaccine induced an increase of these cells in the draining lymph nodes (Figure 2C). Both the mc^2^-CMX and BCG vaccines induced increase of TNF-α^+^ neutrophils in the spleen (Figure 2D).

### Vaccination With mc^2^-CMX Increases the Numbers of NK Cells

**Figure 3:**
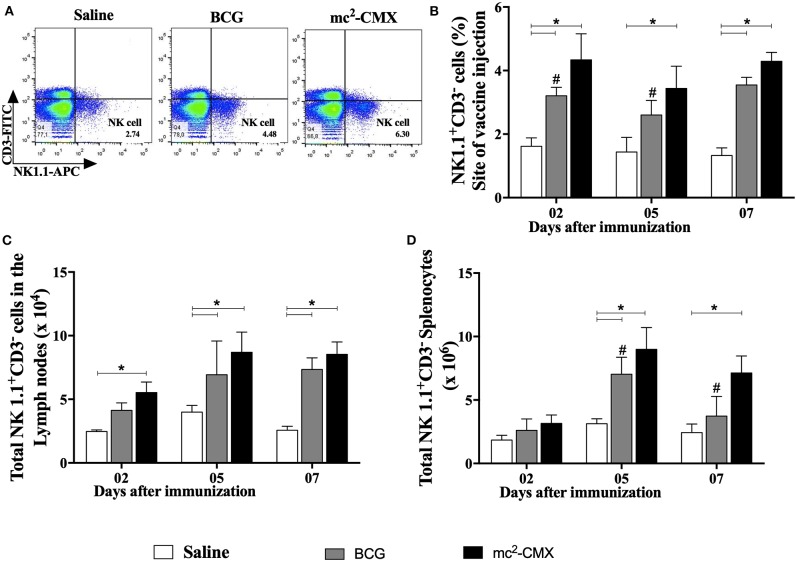
NK cells induced by the mc^2^-CMX and BCG vaccines. C57BL/6 mice were immunized with saline, BCG (10^7^ CFU/mL) or mc^2^-CMX (10^8^ CFU/mL) after 2, 5, and 7 days, the inflammatory lesions generated at the vaccination sites **(B)**, the axillary lymph nodes **(C)** and spleen **(D)** were collected and processed to evaluate the NK cells. **(A)** Representative gates evaluating NK cells (spleen), 7 days after immunization. **(B)** Percentage of NK cells located at the vaccination site. **(C)** Total NK cells in axillary lymph nodes. **(D)** Total NK cells in the spleen. *Statistical difference between vaccinated and the saline groups (*p* < 0.05). ^#^Statistical difference between BCG and mc^2^-CMX groups (*p* < 0.05).

Since neutrophils TNF-α^+^ were increased at the site of vaccination and secondary lymphoid organs, and it has already been shown that these cells may favor NK cell survival and activation (14), we evaluated whether NK cells were present at the site of vaccination and whether these cells were also increased or activated in the draining lymphoid organs (Figure 3). Vaccination with both mc^2^-CMX and BCG induced increase of NK cells at the site of immunization (Figure 3B) as well as in the draining lymph nodes (Figure 3C) and spleen (Figure 3D).

**Figure 4:**
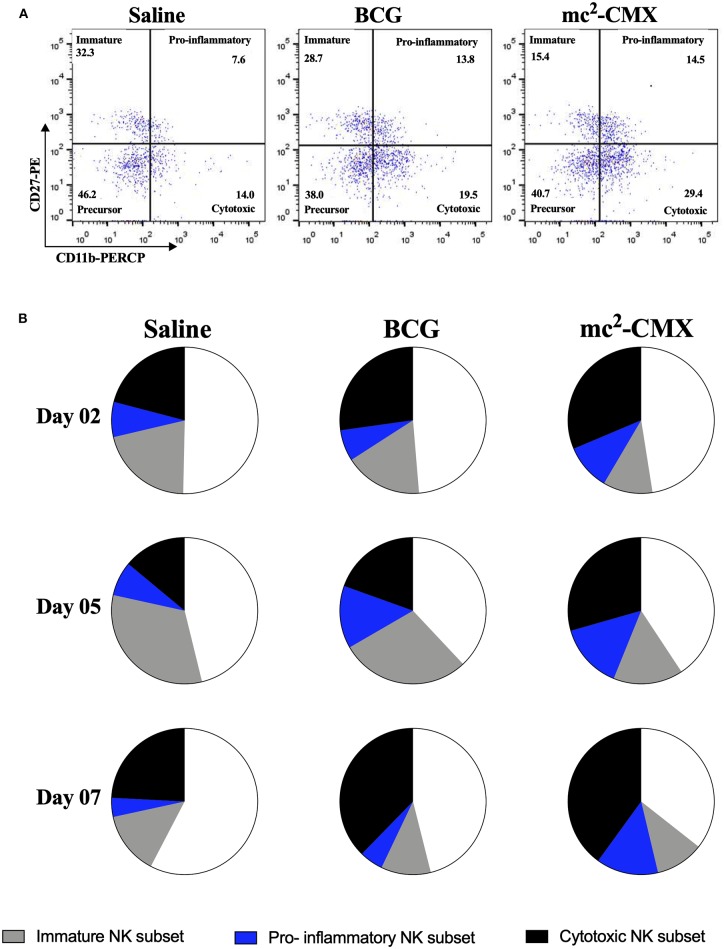
NK cells subpopulations induced by mc^2^-CMX and BCG vaccines in draining lymph nodes. C57BL/6 mice were immunized with saline, BCG (10^7^ CFU/mL) or mc^2^-CMX (10^8^ CFU/mL), and after 2, 5, and 7 days post vaccination, the axillary lymph nodes were collected and processed. **(A)** Representative dot plots quadrants for the determination of the NK cells subpopulations. **(B)** NK cells subpopulations in draining lymph node, 2, 5, and 7 days after vaccination.

As mc^2^-CMX vaccine induced increase of NK cells in the lymph nodes, we investigated the phenotype (subpopulations) of these cells (Figure 4A). It was observed that during vaccination with mc^2^-CMX and BCG there was a predominant increase in cytotoxic NK cell subpopulations (NKc: CD3^−^NK1.1^+^ CD11b^+^ CD27^−^) that accounted for 37.7 and 40.3% of total NK cells, respectively, at day 7. However, twice the percentage of proinflammatory NK cells (NKi; CD3^−^NK1.1^+^ CD11b^+^ CD27^+^) was observed when the animals were vaccinated with mc^2^-CMX (NKi = 13.8%) when compared to BCG group (NKi = 5.1%) at the same time point. Vaccination with mc^2^-CMX also induced greater differentiation of NK cells (NKc + NKi = 54.1%) than vaccination with BCG (NKc + NKi = 42.9%) (Figure 4B). The same NK phenotype changes were observed in the spleen (data not shown).

**Figure 5:**
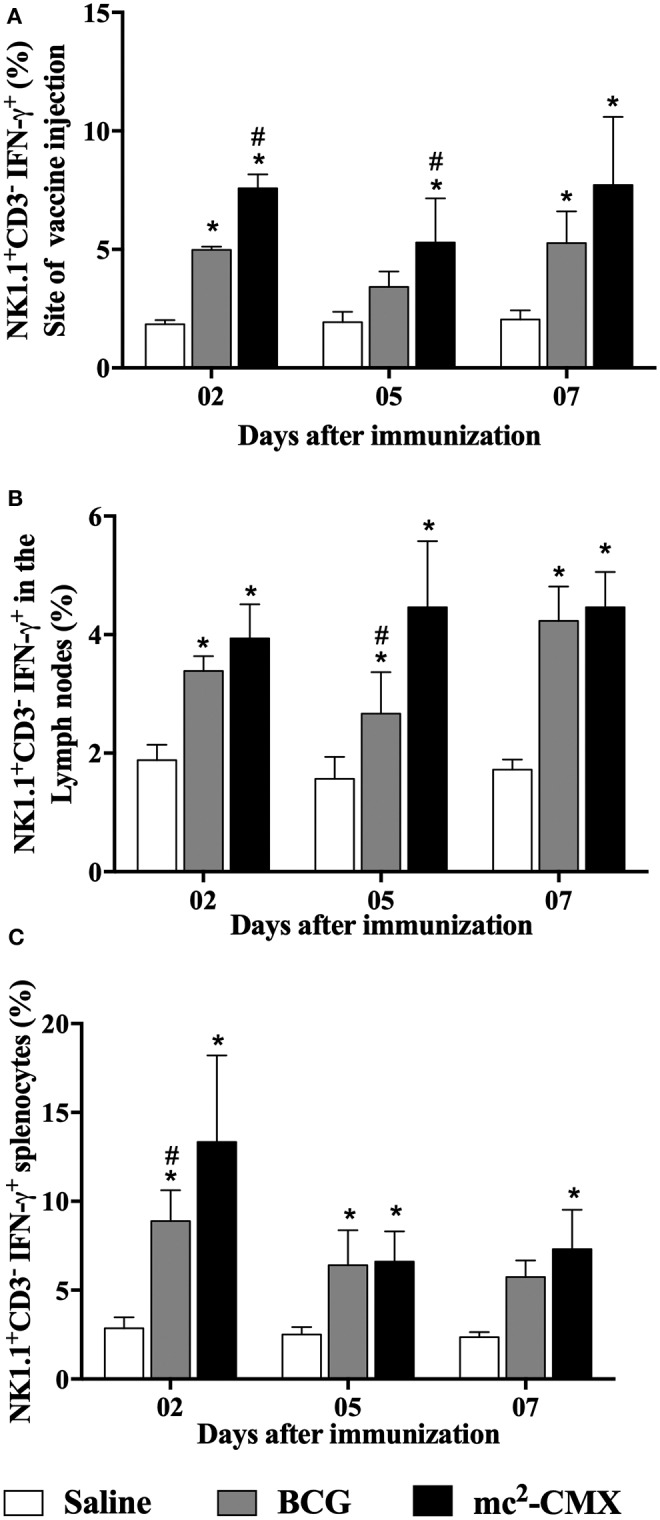
NK^+^IFN-γ^+^ cells induced by mc^2^-CMX or BCG vaccine. C57BL/6 mice (*N* = 5) were immunized with saline, BCG (10^7^ CFU/mL) and mc^2^-CMX (10^8^ CFU/mL) and 2, 5, and 7 days post vaccination, the vaccination site **(A)**, axillary lymph nodes **(B)**, and spleen **(C)** were collected and processed to evaluate NK^+^IFN-γ+ cells. *Statistical difference between the vaccinated groups and the saline group. ^#^Statistical difference between mc^2^-CMX and BCG groups (*p* < 0.05).

Next, we questioned if the increase of proinflammatory NK subpopulation after mc^2^-CMX vaccination could have aided the induction of Th1 specific immune response by expressing IFN-γ, thus NK^+^IFN-γ^+^ cells (CD3^−^NK1.1^+^IFN-γ^+^) were evaluated in the different vaccinated groups. Mice vaccinated with mc^2^-CMX showed higher percentage of NK cells producing IFN-γ than mice from control group, at the vaccination site (Figure 5A), in the draining lymph nodes (Figure 5B) and in the spleen (Figure 5C). Vaccination with mc^2^-CMX induced higher percentage of NK^+^IFN-γ^+^ compared to BCG vaccinated groups in the lymph nodes at 5 days post infection (^#^*p* < 0.05; Figure 5B) and at the site of injection at days 2 and 5 (Figure 5A).

### NK Cell Depletion Does Not Alter the Number of Neutrophils or Dendritic Cells After the Vaccination With mc^2^-CMX

**Figure 6:**
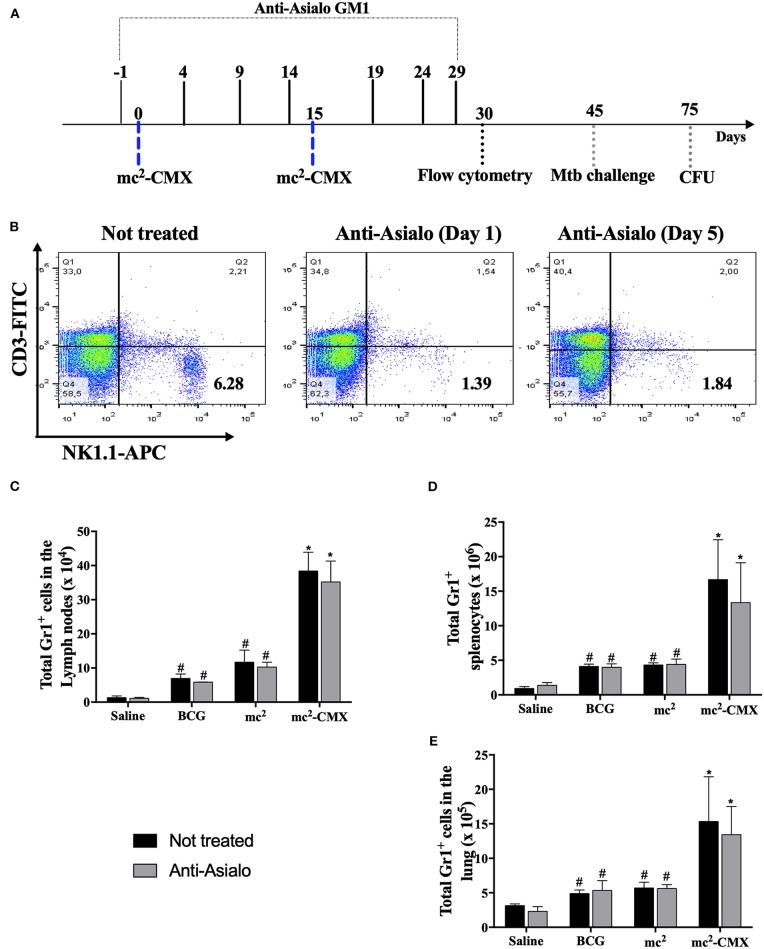
NK cell depletion. C57BL/6 mice were treated with anti-asialo antibody and after the first vaccination at 1- or 5-days post depletion the spleens were collected and checked for NK cell depletion **(A)**. Representative flow cytometry showing depletion induced by the anti-Asialo GM1 antibody in the spleen **(B)**. **(C–E)** C57BL/6 mice (*N* = 5) after NK cell depletion were immunized with saline, BCG (10^7^ CFU/mL), mc^2^ (10^8^ CFU/mL), or mc^2^-CMX (10^8^ CFU/mL). After 15 days of the last vaccination with mc^2^-CMX, the mice were euthanized, and axillary lymph nodes, spleen, and lungs were collected for evaluation of the presence of neutrophils (Gr1^+^ cells) by flow cytometry. **(A)** Total neutrophils present in the axillary lymph nodes. **(B)** Total neutrophils present in the spleen. **(C)** Total neutrophils present in the lung. *Statistical difference between untreated groups and vaccinated groups. ^#^Statistical difference between groups and mc^2^-CMX vaccinated groups (*p* < 0.05).

Since mc^2^-CMX vaccination induced a greater number of NK cells with a proinflammatory phenotype, it was investigated whether those cells influenced neutrophil or DC migration. For that, C57BL/6 mice were treated with anti- ganglioside (ganglion-N-tetraoxyl ceramide) antibody (anti-asialo antibody; Figure 6A) which resulted in about 80% of the NK cell population depletion, as shown in Figure 6B. NK cell depletion did not affect any change on the number of neutrophils induced by any vaccine treatment 31 days after initiation of anti-asialo treatment (Figure 6). Thus, mc^2^-CMX or BCG vaccination continued to induce an increase in neutrophil numbers in the draining lymph nodes (Figure 6C), spleen (Figure 6D), and lung (Figure 6E) regardless of the presence of NK cells.

**Figure 7:**
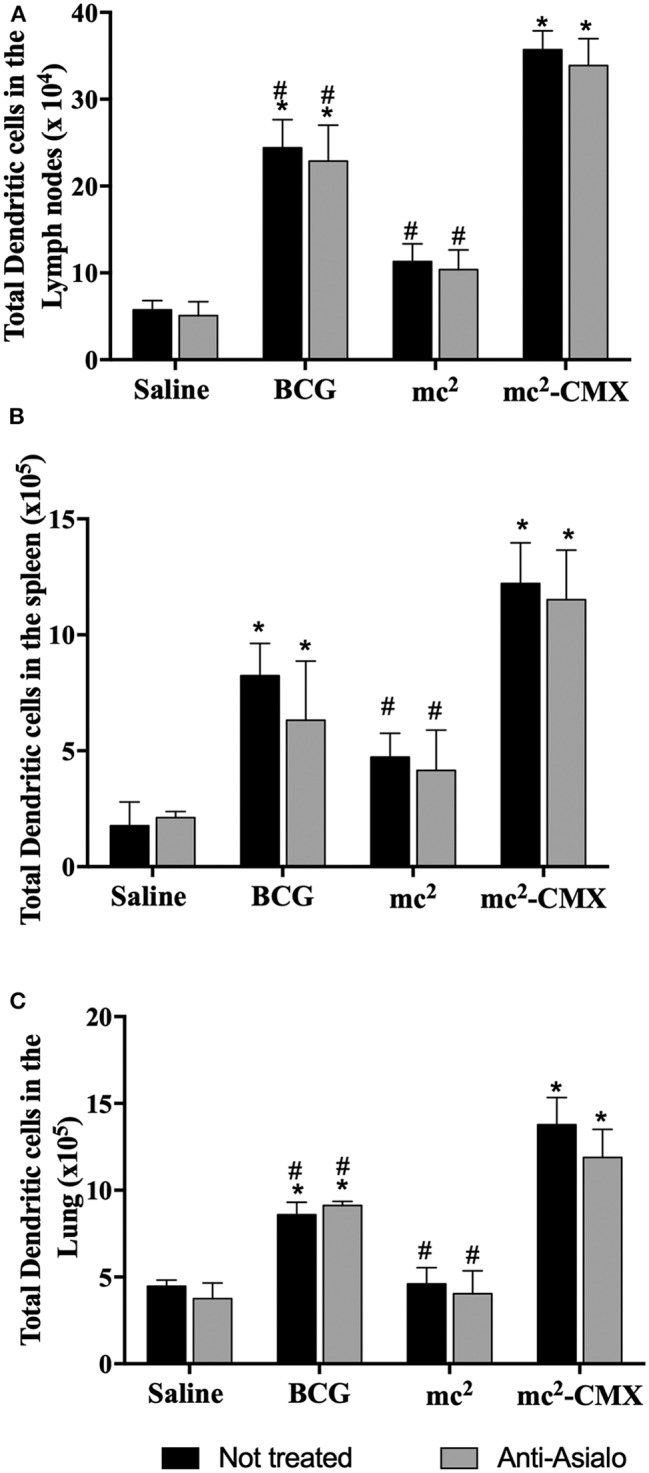
Dendritic cell increase due to mc^2^-CMX vaccination is NK cells independent. C57BL/6 mice (*N* = 5) depleted of NK cells were immunized with saline, BCG (10^7^ CFU/mL), mc^2^ (10^8^ CFU/mL), or mc^2^-CMX (10^8^ CFU/mL). Fifteen days after the last vaccination with mc^2^-CMX, mice were euthanized, and axillary lymph nodes **(A)**, spleen **(B)**, and lungs **(C)** were collected and evaluated for the presence of dendritic cells (CD11c^+^ cells) by flow cytometry. *Statistical difference between groups and mc^2^-CMX vaccinated groups. ^#^Statistical difference between the group vaccinated with mc^2^-CMX and the other groups (*p* < 0.05).

**Figure 8:**
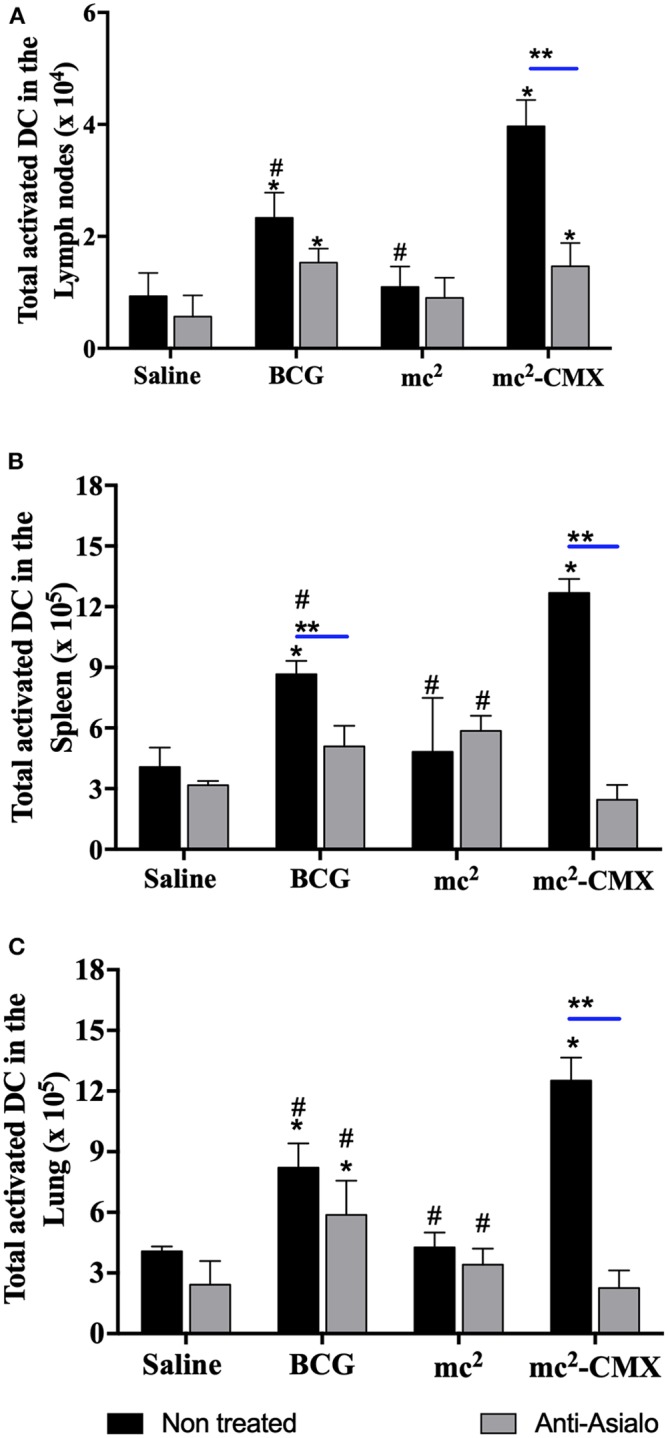
NK cells depletion reduces DC activation after vaccination with mc^2^-CMX. C57BL/6 mice (*N* = 5) depleted or not of NK cells were immunized with saline, BCG (10^7^ CFU/mL), mc^2^ (10^8^ CFU/mL), or mc^2^-CMX (10^8^ CFU/mL). Fifteen days after the last vaccination, mice were euthanized, and axillary **(A)**, spleen **(B)**, and lung lymph nodes **(C)** were collected for evaluation of total activated dendritic cells (CD11C^+^MHCII^+^CD11b^+^) by flow cytometry. *Statistical difference between groups and mc^2^-CMX vaccinated groups. ^#^Statistical difference between the group vaccinated with mc^2^-CMX and the other groups (*p* < 0.05). **Statistical difference between anti-asialo treated and untreated group (*p* < 0.05).

As NK cells can aid DCs in the generation of a specific immune response, it was evaluated whether the absence of these cells could alter the proportion of DCs generated by the vaccines. Figure 7 shows that, in the absence of NK cells, the number of DCs did not change, and vaccination with mc^2^-CMX or BCG induced an increase of these cells in lymph nodes (Figure 7A), spleen (Figure 7B), and lungs (Figure 7C), when compared with saline. mc^2^-CMX vaccination presented at this time point, higher levels of DC in all evaluated organs. Although, in the absence of NK, the number of DC did not change, their activation status could have been modified. That was indeed the case, as shown in Figure 8 for all evaluated organs, activated DC were reduced upon NK cell depletion among mc^2^-CMX vaccinated animals. NK depletion mainly affected DC activation in the spleen (Figure 8B). Thus, NK cells could be important for the activation of dendritic cells, which may be crucial in inducing a specific immune response to vaccines.

### Absence of NK Cells Reduce the Specific Immune Response Generated by mc^2^-CMX Vaccination

**Figure 9:**
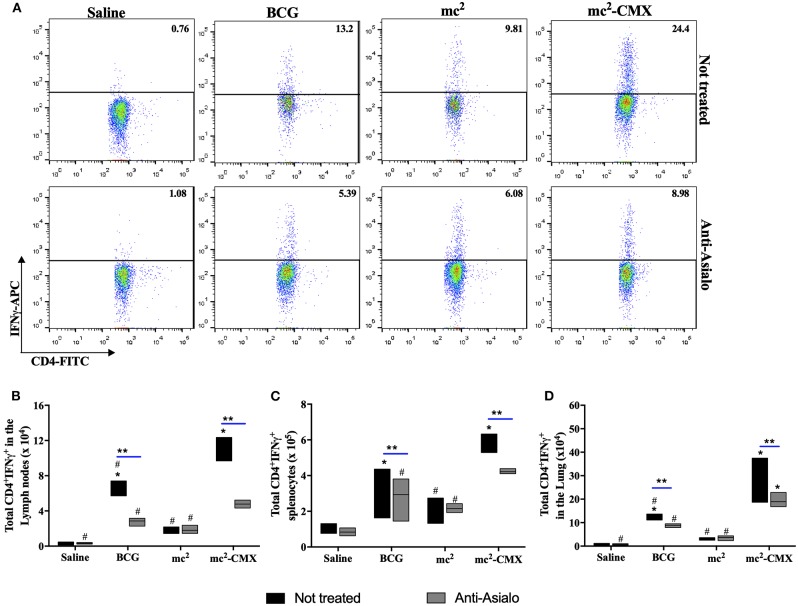
NK cells depletion reduces the number of specific Th1 lymphocytes induced by vaccination. C57BL/6 mice (*N* = 5) depleted or not of NK cells were immunized with saline, BCG (10^7^ CFU/mL), mc^2^ (10^8^ CFU/mL), or mc^2^-CMX (10^8^ CFU/mL). Fifteen days after the last vaccination with mc^2^-CMX, axillary lymph nodes, spleen and lung were collected, and their cells were processed and incubated with medium (control without stimulation), ConA (1 μg) or CMX (1 μg) for specific CMX Th1 cells evaluation. **(A)** Representative dot plots of CD4^+^IFN-γ^+^ T cells. **(B)** Total number of CD4^+^IFN-γ^+^ T cells in the axillary lymph nodes. **(C)** Total of CD4^+^IFN-γ^+^ T cells in the spleen. **(D)** Total CD4^+^IFN-γ^+^ T cells in the lungs. *Statistical difference between groups and mc^2^-CMX vaccinated groups. ^#^Statistical difference between the group vaccinated with mc^2^-CMX and the other groups (*p* < 0.05). **Statistical difference between anti-asialo treated and untreated group (*p* < 0.05).

Because the absence of NK cells reduced the number of activated DCs after vaccination with mc^2^-CMX, generation of the specific Th1 immune response (CD4^+^IFN-γ^+^ T cells) could also be altered. In Figure 9, it is observed that in the absence of NK cells, after stimulation with recombinant CMX protein, the number of Th1 lymphocytes was reduced after vaccination with either mc^2^-CMX or BCG. However, it was noted that despite the reduction of specific Th1 cells, vaccination with mc^2^-CMX still induced greater numbers of CD4^+^IFN-γ^+^ T cells in the lymph nodes (Figure 9A) and in the lungs (Figure 9D) when compared with BCG.

### Absence of NK Cells Reduce the Protection Conferred by mc^2^-CMX Vaccination

**Figure 10:**
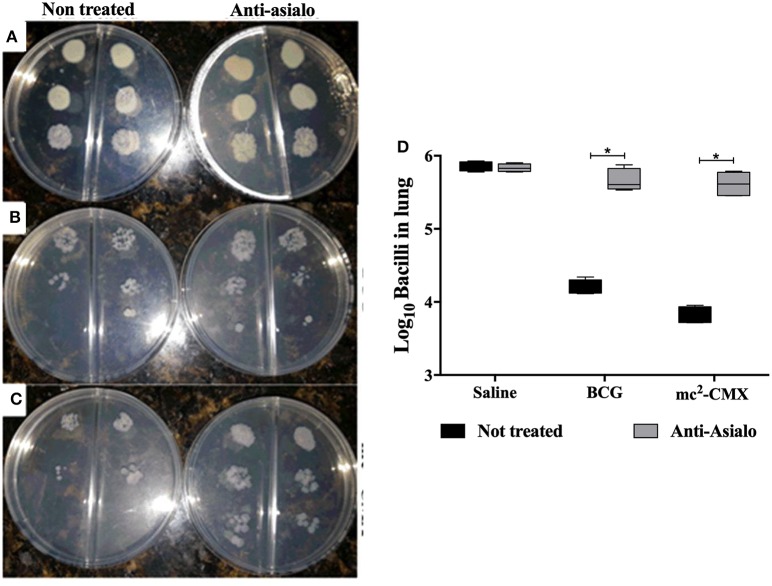
NK cell depletion impairs protection generated by mc^2^-CMX or BCG vaccination. C57BL/6 mice (*N* = 5) depleted or not were immunized with saline, BCG (10^7^ CFU/mL), mc^2^ (10^8^ CFU/mL), or mc^2^-CMX (10^8^ CFU/mL). Fifteen days after the last vaccination with mc^2^-CMX, animals were challenged with *M. tuberculosis* and 30 days after challenge, lungs were collected, processed, homogenized and plated to determine the bacillary load. **(A–C)** dilutions 1/100, 1/1,000, and 1/10,000 were plated and cultured for 28 days. **(D)** Bacillary load in the lungs of infected mice. *Statistical difference between not depleted groups.

The absence of NK cells reduced the number of specific CD4^+^IFN-γ^+^ T cells induced by vaccination with mc^2^-CMX, thus it was enquired whether this could influence the protection generated by the vaccination. The bacillary load was evaluated 30 days after infection with *M. tuberculosis* and while NK cells depletion did not influence the bacterial load in naïve mice (Figures 10A,D) as our group have previously shown (7) the protection generated by either BCG or mc^2^-CMX vaccines was significantly diminished in the absence of NK cells (Figures 10B–D). These results suggest that NK cells are important for vaccine Th1 cells generation and required for the protection induced by mc^2^-CMX and BCG vaccines.

### Influence of NK Cells or Neutrophils on Their Migration in Response to Vaccine

**Figure 11:**
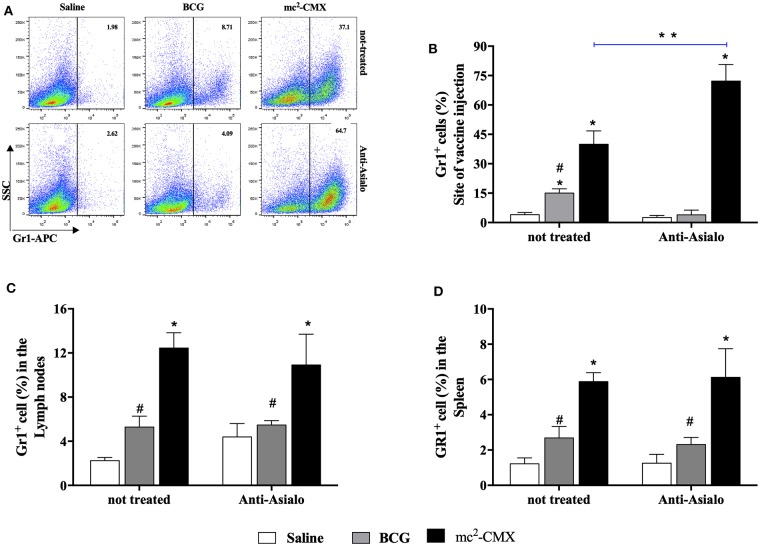
NK cells absence does not interfere with migration of neutrophils cells after vaccination with mc^2^-CMX. C57BL/6 mice (*N* = 5) treated or not with anti-Ly6G anti-asialo were immunized with saline, BCG (10^7^ CFU/mL), or mc^2^-CMX (10^8^ CFU/mL). After 5 days of the last vaccination, the mice were euthanized, and axillary lymph nodes, spleen, and the tissue at the site of vaccination were collected for evaluation of the presence of NK cells (CD3^−^NK1.1^+^). **(A)** Representative dot plot of neutrophils gate strategy (GR-1^+^). **(B)** Percentage of GR-1^+^ cells at the site of vaccine injection after NK depletion. **(C)** Percentage of GR-1^+^ cells in the lymph nodes after NK depletion. **(D)** Percentage of GR-1^+^ cells in the spleen after NK depletion. *Statistical difference between control groups. **Statistical difference between the group (vaccinated with the same vaccine) depleted and not depleted (*p* < 0.05). ^#^Statistical difference between anti-asialo treated group and mc2-CMX anti-asialo treated group (*p* < 0.05).

**Figure 12:**
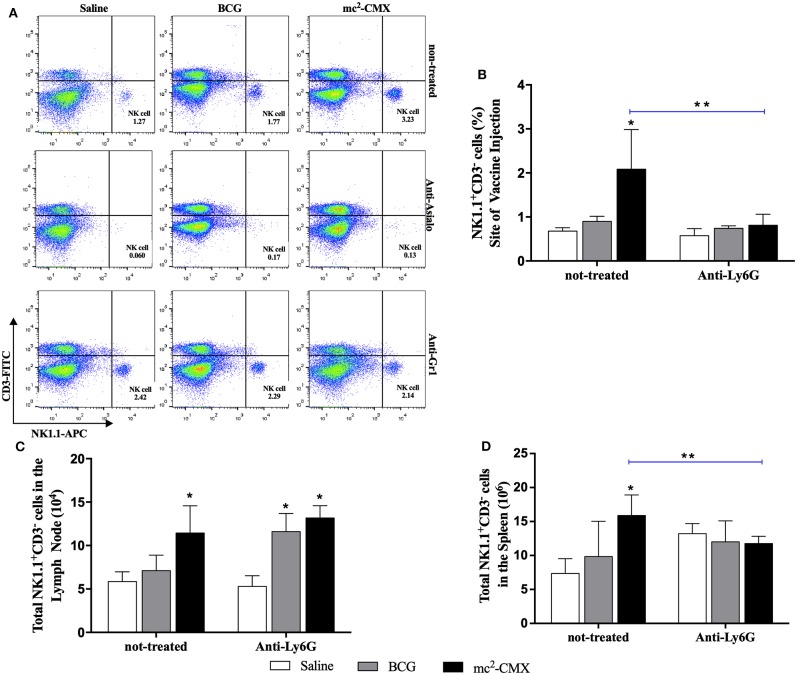
Neutrophils depletion interfere with migration of NK cells after vaccination with mc^2^-CMX. C57BL/6 mice (*N* = 5) treated or not with anti-GR-1 were immunized with saline, BCG (10^7^ CFU/mL), or mc^2^-CMX (10^8^ CFU/mL). After 5 days of the last vaccination, the mice were euthanized, and axillary lymph nodes, spleen, and the tissue at the site of vaccination were collected for evaluation of the presence of NK cells (NK1.1^+^CD3^−^). **(A)** Representative dot plot of NK gate strategy (NK1.1^+^CD3^−^) during anti-asilo treatment and anti-GR-1 treatment in the lymph nodes. **(B)** Percentage of NK cells at the site of vaccine injection after neutrophil depletion. **(C)** Percentage of NK cells in the lymph nodes after neutrophil depletion **(D)** Percentage of NK cells in the spleen after neutrophil depletion. *Statistical difference between control groups. **Statistical difference between the group (vaccinated with the same vaccine) depleted and not depleted (*p* < 0.05).

To evaluate the influence of neutrophils on NK cells migration/presence at the site of vaccine injection, or vice versa, mice were vaccinated during each cell population depletion, and the presence of neutrophils or NK cells were investigated at the site of vaccination, lymph nodes and spleens. When NK cells were depleted, the number of neutrophils at the site of vaccination increased even further than in non-depleted group (Figures 11A,B) and maintained with higher levels in the lymph node and spleen (Figures 11C,D). Depletion of neutrophils resulted in reduction on the number of NK cells at the vaccination site and spleens to the same level of the non-vaccinated saline group (Figure 12).

## Discussion

In this work, it was possible to observe that mc^2^-CMX and BCG vaccination resulted in increased number of neutrophils as well as cytotoxic and pro-inflammatory NK cells, when compared to the saline vaccinated group. In addition, in absence of NK cells there was a reduction in the number of activated DCs and specific Th1 cells generated by the mc^2^-CMX and BCG vaccinations, which culminated in the reduction of the protection against TB, generated by this vaccine.

After immunization, a high number of neutrophils were found at the immunization site and in lymphoid organs and those neutrophils were also able to produce TNF-α^+^. Although we cannot attest that the neutrophils found in the secondary lymphoid organs, after vaccination with mc^2^-CMX, had been drained from the immunization site directly impacting on the vaccine immune response development, Abadie et al. (29) showed that neutrophils, after phagocytizing the intradermally applied BCG, migrated to the draining lymph nodes, therefore we believe that the neutrophils increase, in the organs evaluated here, were associated in part with the migration from the inoculation site. Additionally, Blomgran and Ernst (30) demonstrated that DCs, which phagocyted neutrophil apoptotic bodies, are more prone to migrate and induce Th1 response. Bennouna et al. (31) demonstrated that TNF-α from neutrophils aided DCs and macrophages differentiation and activation, and probably are essential to initiate a protective cellular immune response against Mtb. However, Bekker et al. (32) showed that the susceptibility to BCG depends on the TNF-α levels, where lower levels of that cytokine were correlated to constrict granulomas and better protection, while excessive levels were associated with excessive inflammatory responses. We showed before that, in the absence of neutrophils, the protection induced by vaccination with mc^2^-CMX was reduced, but neutrophil depletion did not interfere with the protection induced by BCG (27). Here, the observation that the levels of neutrophils and TNF-α^+^ neutrophils induced by BCG vaccination were inferior to the ones induced by mc^2^-CMX vaccine at the site of vaccination favors the argument that TNF-α^+^ neutrophils are crucial for a better protection against Mtb challenge only for mc^2^-CMX as showed before.

The mycobacterial cell wall components activate neutrophils and induce the production of cytokines such as TNF-α, IL-6, and IL-8 (33, 34). It has been shown that *Mycobacterium smegmatis*, the bacillus used here to express CMX, induces greater release of inflammatory cytokines by neutrophils when compared to *Mycobacterium tuberculosis* (35). Perhaps this fact justifies the greater migration of TNF-α^+^ neutrophils in animals vaccinated with the mc^2^-CMX vaccine when compared to BCG vaccinated animals. When the immune response generated by mc^2^-CMX or BCG vaccines was evaluated, using BALB/c mice as model, it was observed that the mc^2^-CMX vaccine had a higher induction of specific CD4^+^IFN-γ^+^ and CD4^+^TNF-α^+^ T cells than BCG (36). Here, using C57BL/6 mice, mc^2^-CMX also presented higher levels of CD4^+^IFN-γ^+^ T cells in Lymph nodes, spleens and lungs (Figure 9) than BCG vaccinated mice. Although we did not evaluate here, the induction of Th17 cells, responsible for activation and migration of neutrophils present mainly upon vaccination with mc^2^-CMX, appeared to be the foremost responsible for this vaccine protection (25).

The vaccination with mc^2^-CMX and BCG induced NK cell recruitment and maturation. The maturation of cytotoxic NK cells occurred for both mc^2^-CMX and BCG vaccination, and that can be explained by mycobacteria cell wall components (such as mycolic acid, peptideoglycan and arabinomannan), which activate NK cells via TLR-2. This activation improves cytotoxic function of NK cells through receptors, such as NKp44, NKp46, and NKG2D (37, 38). Although, in this work we did not evaluate NK receptors, we believe that they could be involved in this activation, since mycolic acid among other Mtb molecules can be recognized by NKp44 (38).

The vaccines tested in this work induced NKi, and vaccination with mc^2^-CMX doubled the amount of those cells when compared to BCG vaccine. NKi population was described as being capable of secreting proinflammatory cytokines (such as IFN-γ) that activate macrophages, by inducing migration and improving phagocytic and oxidative function (8, 39), thus indirectly culminating in Mtb clearance (40) and indirectly generating CD4^+^IFN-γ^+^ T cells. This may have an impact on Mtb vaccine protectiveness effect, once NKi cells could be important for Th1 lymphocyte differentiation by activated DCs (41). Here, we cannot directly correlate the NKi cells that were induced by the vaccinations with the CD4^+^IFN-γ^+^ T cells accumulation and protection, but mc^2^-CMX vaccine induced higher levels of those cells when compared with BCG, and NK depletion during vaccination culminated with excessive decrease of those cells that abrogated protection for both vaccines. It is important to note that NK depletion during mc^2^-CMX vaccination, although causing CD4^+^IFN-γ^+^ T reduction in the lungs, the total numbers of this population was higher than the saline control groups suggesting that other cells are also involved in the generation of Th1 cells and protection.

The interaction of NK cells with neutrophils may induce apoptosis of neutrophils dependent of NKp46, Fas and NKG2D (21, 42) and has been suggested, for BCG vaccination, that these apoptotic bodies are transported by the DCs cells to the lymph nodes and are responsible for the induction of the specific response to BCG (29). Here, when NK cells were depleted during mc^2^-CMX vaccination, it was observed an increase in the neutrophil population in the evaluated organs indicating some type of modulation by NK-neutrophil interaction occur. It is interesting to note that neutrophil depletion does not affect NK cell population during BCG vaccination (3), but vaccination with mc^2^-CMX drastically reduce the NK levels induced by immunization. We argue that BCG vaccination might involve a tight NK-Neutrophils-Th1 response that does not need to modulate the neutrophil numbers, while during mc^2^-CMX vaccination, for which protection depends also on Th17 cells, NK cells might also produce immunosuppressive cytokines (9) to modulate both Th1 and Th17 cells to produce a balanced immune response.

Regarding the DCs number and activation, mc^2^-CMX vaccination increased the activation and the number of DCs in all evaluated organs when compared to BCG. Studies have shown that the interaction between NK cells and DCs induces the production of IFN-γ and TNF-α by NK cells, and IL-12, IL-15, or IL-18 by DCs (43, 44). It has also been shown that NK cells are important for the development of the specific Th1 response (10, 15). These functions of both NK and dendritic cells may explain our results, where in the absence of NK cells there was a reduction in the DC activation that culminated with reduction of specific Th1 cells. Although, Dihman et al. (3) observed a similar result for Th1 cells when BCG vaccine was used and NK cells were depleted, they did not evaluate DC activation. Here we showed that only splenic DCs presented reduced activation upon NK depletion, indicating that another cells/cytokine/chemokine might be activating DC besides NK and its cytokines. In our vaccine model (mc^2^-CMX), NK cells, neutrophils, and dendritic cells seem to be acting together, with the vaccine inducing increase of cytotoxic NK subset, which in turn might leads to apoptosis of neutrophils harboring phagocytosed mc^2^-CMX (at vaccination site), that could result in the release of apoptotic bodies that activate DCs, which could present the antigens and generate an adaptive immune response.

The non-specific effects of BCG vaccination are widely known in literature, for instance the NK cells training immunity in BCG vaccination shown by Kleinnijenhuis et al. (45) demonstrated that NK cells induced by BCG were important in the protection against Mtb and other microorganisms. Here, mc^2^-CMX induced NK cells maturation, however we didn't evaluate the training effect of our vaccine in these cells, and this could be a matter of future investigation.

We and others have been using the intravenous route to challenge mice, which, despite not representing the natural route of infection, allows us to work safely and assured that the pathogenesis induced by the intravenous and aerosol routes are similar (46, 47). Although these results were observed using vaccines composed of two different strains of mycobacteria at two different doses, we cannot exclude the interference of the bacterial load in generation of these results.

In conclusion, the protective immune response induced by BCG vaccine does not depend on neutrophils, while the protective response elicited by mc^2^-CMX depends on neutrophils and NK cells. Neutrophil depletion directly affects the NK cell population induced by mc^2^-CMX, while in the absence of NK, DC activation is reduced but the number of neutrophils is not affected. Future work should be done in order to understand the mechanisms of NK and neutrophils cooperation.

## Data Availability Statement

The datasets generated for this study are available on request to the corresponding author.

## Ethics Statement

The animal study was reviewed and approved by Comitê de Ética em Pesquisa no Uso de animais-CEUA/UFG.

## Author Contributions

AJ-K conceived the idea, supervised the experiments, evaluated the results, and wrote the final version of the manuscript. MT and LM did the animal experiments and wrote the first draft of the manuscript. AK critically revised the results and manuscript. All authors discussed the results and agreed with the results and ideas as presented.

## Conflict of Interest

The authors declare that the research was conducted in the absence of any commercial or financial relationships that could be construed as a potential conflict of interest.
